# Identification of Region-Specific Myocardial Gene Expression Patterns in a Chronic Swine Model of Repaired Tetralogy of Fallot

**DOI:** 10.1371/journal.pone.0134146

**Published:** 2015-08-07

**Authors:** Sabine Charron, François Roubertie, David Benoist, Virginie Dubes, Stephen H. Gilbert, Marion Constantin, Delphine Vieillot, Delphine Elbes, Bruno Quesson, Pierre Bordachar, Michel Haissaguerre, Olivier Bernus, Jean-Benoit Thambo, Caroline Rooryck

**Affiliations:** 1 L’Institut de Rythmologie et Modélisation Cardiaque LIRYC, Université de Bordeaux, Pessac, France; 2 Inserm U1045 CRCTB, Université de Bordeaux, Bordeaux, France; 3 Hôpital cardiologique Haut-Lévêque, CHU de Bordeaux, Pessac, France; 4 Max Delbrück Center for Molecular Medicine, Berlin, Germany; 5 Plateforme Technologique d’Innovation Biomédicale, Université de Bordeaux, Pessac, France; 6 University of Oxford, Institute of Biomedical Engineering, Oxford, United-Kingdom; 7 Laboratoire Maladies Rares: Génétique et Métabolisme (MRGM), EA 4576, Université de Bordeaux, Bordeaux, France; Northwestern University, UNITED STATES

## Abstract

Surgical repair of Tetralogy of Fallot (TOF) is highly successful but may be complicated in adulthood by arrhythmias, sudden death, and right ventricular or biventricular dysfunction. To better understand the molecular and cellular mechanisms of these delayed cardiac events, a chronic animal model of postoperative TOF was studied using microarrays to perform cardiac transcriptomic studies. The experimental study included 12 piglets (7 rTOF and 5 controls) that underwent surgery at age 2 months and were further studied after 23 (+/- 1) weeks of postoperative recovery. Two distinct regions (endocardium and epicardium) from both ventricles were analyzed. Expression levels from each localization were compared in order to decipher mechanisms and signaling pathways leading to ventricular dysfunction and arrhythmias in surgically repaired TOF. Several genes were confirmed to participate in ventricular remodeling and cardiac failure and some new candidate genes were described. In particular, these data pointed out *FRZB* as a heart failure marker. Moreover, calcium handling and contractile function genes (*SLN*, *ACTC1*, *PLCD4*, *PLCZ*), potential arrhythmia-related genes (*MYO5B*, *KCNA5*), and cytoskeleton and cellular organization-related genes (*XIRP2*, *COL8A1*, *KCNA6*) were among the most deregulated genes in rTOF ventricles. To our knowledge, this is the first comprehensive report on global gene expression profiling in the heart of a long-term swine model of repaired TOF.

## Introduction

Tetralogy of Fallot (TOF) is the most frequent cyanotic congenital heart disease in humans with an incidence of 1/3600 live births [1]. Surgical early primary repair is highly successful and the population of adults with repaired Tetralogy of Fallot is increasing rapidly. However, the long-term outcome of these patients may be complicated by right ventricular [RV] or biventricular dysfunction and by sudden cardiac death (around 5%). The incompletely understood mechanisms of these delayed events may partially be due to surgically-induced permanent right bundle branch block (BBB) and ventricular dyssynchrony [2,3,4]. Unlike left ventricular failure, RV failure is poorly understood and its management remains largely empirical. In particular, the molecular mechanisms underlying different stages of ventricular remodeling and its progression towards heart failure (HF) remain obscure. Indeed, a recent position paper from the ESC working Group on Myocardial Function have emphasized the interest of working on the functional and molecular changes occurring in the right ventricle [5].

In order to better understand the implication of prolonged dyssynchrony, we established and characterized a chronic large animal model that mimicked essential parameters of postoperative TOF [6]. This model represents a reliable long-term swine model of RV dysfunction and dyssynchrony, with echocardiographic measurements comparable to adult patients with early surgical repaired TOF. Indeed, large animal models have a closer physiology to humans than small mammal models and are more appropriate to study the pathophysiological changes associated with ventricular remodeling.

Moreover, in the past decade, the completion of Sus Scrofa genome sequencing (Sscrofa10.2, INSDC Assembly, Aug 2011) led to the development of commercially available pig cDNA microarrays that could be used to detect changes in swine genes expression.

In the present study, we aimed at deciphering the sequence of molecular events and pathways leading to the progression of ventricular dysfunction and arrhythmias, by studying gene expression profiles of repaired Fallot (rTOF) pigs’ hearts compared to control animals (sham-operated), in four different localizations of the heart (epicardium and endocardium of right and left ventricles), by a whole genome approach. Some transcripts were selected to verify the accuracy and reproducibility of the microarray data by real-time qRT-PCR.

## Materials and Methods

The experimental protocol followed the European rules for animal experimentation (European legislation 2010/63/UE—2010) which was implemented under French legislation from February 2013 and following which all animal experimental protocols, including the present study, at our Institution were reviewed and approved by the local Ethics Committee "Comité d'Ethique en Expérimentation Animale de Bordeaux—CEEA50. The experimental protocols were in compliance with the Guiding Principles in the Use and Care of Animals published by the National Institutes of Health (NIH Publication No. 85–23,Revised 1996).

### rTOF swine model

The experimental study included 12 piglets (7 rTOF and 5 controls), aged three months and weighing less than 12 kg, at the time of surgery. The animal model was established as previously described [6]. Briefly, after left thoracotomy of seven piglets, the RV outflow tract was partially occluded with a clamp and incised longitudinally across the pulmonic valve annulus. The operation was designed to cause RV volume overload from valvular regurgitation by excision of two pulmonic valve leaflets, RV pressure overload by a loose tape partially occluding the pulmonary artery, and RV outflow tract scar around the patch placed to close the RV incision. Five piglets were sham operated to serve as controls. After the procedure was completed, the animals were extubated and received supplemental oxygen and analgesia as needed, before their transfer to a long-term postoperative care facility. After intervention, qualitative cardiac evaluation of the pigs was performed by echocardiogram and color Doppler in order to confirm the pulmonary regurgitation in the rTOF pigs.

After 23 (+/- 1) weeks of postoperative recovery, cardiac function was assessed in anesthetized pigs (isoflurane 2%, Vibrac) by cardiac magnetic resonance in a Siemens Magnetom Avanto 1.5T MRI scanner (Erlangen, Germany). Animals were euthanized, their hearts excised and washed in ice-cold cardioplegic solution to remove any residual blood.

### Tissue collection and RNA extraction

Myocardial samples were dissected out and immediately frozen into liquid nitrogen. Biopsies were then stored at -80°C until RNA extraction. Total RNA was extracted from tissues using QIAzol reagent (Qiagen, USA). RNA was purified and DNase treated using the QIAGEN RNeasy Kit. RNA purity and integrity, were assessed both by spectrophotometry (NanoDrop ND-1000, NanoDrop Technologies) and nanoelectrophoresis (2100 Bioanalyzer, Agilent Technologies).

### Microarray Hybridization and scanning

cDNA was synthesized from 200 ng of total-RNA using the direct cDNA Labeling System. Aminoallyl-cRNA was synthesized from cDNA using the Superscript Indirect cDNA Labeling System. The cRNA was purified using RNeasy QIAGEN RNeasy Kit.

Labeling and hybridization of the cRNA was performed with Agilent Whole Porcine Genome Oligo (4 × 44 K) Microarrays (one-color platform), according to the manufacturer’s protocols. The slides were scanned and analyzed using the histogram method with default settings in an Agilent G2565C Microarray Scanner System with SureScan Technology.

### Annotation of the porcine microarray

Among the 43,603 probes present on the Porcine 44K Agilent microarray (V2), only 15,458 of these were annotated with HUGO Gene Nomenclature Committee (HGNC) gene symbols [7], corresponding to about 7600 genes. This incomplete annotation was probably due to the presence of former ESTs (Expressed Sequence Tags) that no longer belong to Sus Scrofa after the completion of the pig genome sequencing project. More probes were annotated using GeneBank and Basic local Alignment Search tool program (BLAST, NCBI).

### Microarray Data Analysis

Hierarchical clustering of the significantly differentially expressed (DE) genes from microarray data was carried out by the Genespring software (Agilent Technologies), with a p-value corrected by Benjamini Hochberg False Discovery Rate (FDR). Different softwares were used to interpret the biological functions and canonical pathways of the gene lists: Visualization and Integrated Discovery Online platform (http://david.abcc.ncifcrf.gov/)[8], using Gene ontology (GO) terms and the Database for Annotation, with a threshold of a minimum three genes annotated at each node, Panther Pathway (http://www.pantherdb.org/pathway/) and Ingenuity Pathway Analysis (IPA, http://www.ingenuity.com)

### Reverse Transcription quantitative PCR (RT-qPCR)

In order to confirm the reliability of the expression profile from the microarray analyses, the expression level of some genes was assessed by real-time RT-qPCR.

Sequences for primers were obtained from Ensembl Genome Browser. Primers were designed using Primer designing tool (NCBI) and synthesized at Sigma Aldrich. 1μg of RNA was reversed transcribed using a cDNA Reverse Transcription kit (Life Technologies) according to the manufacturer’s protocol. Quantitative PCR was performed in a 25 μL reaction volume (2 μL cDNA, 12,5 μL of SYBR Premix (BIO-RAD), a volume of 10 μM upstream and downstream primers respectively, and added ddH2O to 25 μL) on the BIO-RAD C 100 Touch Thermal Cycler / CFX96 Real time System. Real-time PCR conditions were as follows: 3 min at 95.0°C, 40 cycles of denaturation at 95°C for 30 s followed by 30 s annealing and elongation at 60°C. Efficiency of primer pairs was previously evaluated. Melting curves were obtained at the end of each run to confirm a single PCR product. All samples were run in triplicate. Non-template controls were included in each run to exclude contamination and nonspecific amplification. Expression levels of samples were normalized by using a normalization factor calculated by the software CFX Manager (BIO-RAD). This normalization factor was calculated based on RT-qPCR results for two selected reference genes, *HPRT1* and *GUSB*. This allowed quantification of the target gene in one sample relative to that in another (the calibrator) using the “2−ΔΔCt method” of calculating fold changes in gene expression.

## Results

Upon cardiac magnetic resonance examination at 23 ± 1 weeks, Fallot pigs presented with significantly decreased RV ejection fraction and increased RV end-diastolic volume compared to controls indicating RV dysfunction and RV dilation respectively(S1 Fig).

We first performed microarray experiments on myocardial biopsies from the right ventricle (endocardium and epicardium separately), because we expected more changes in this particular region with an animal model of right ventricular dysfunction. We observed the biggest list of statistically significant DE genes in the endocardium of the right ventricle and focused on the exploration of these genes. We then explored the left ventricle (endocardium and epicardium).

### Microarray Profiling of the endocardium of the right ventricle

**Fig 1:**
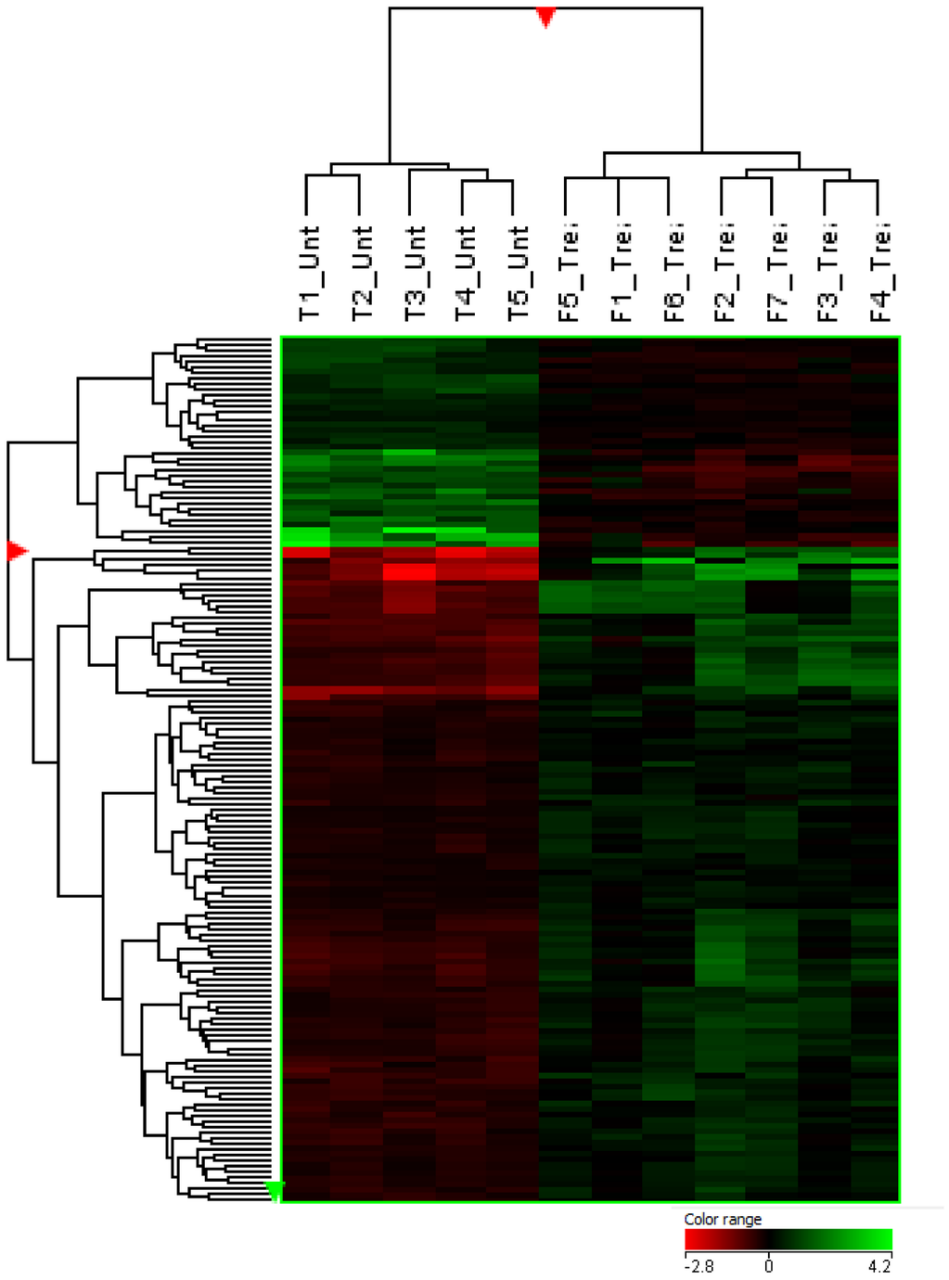
Heatmap displaying the 153 most differentially expressed probes (p<0.05 FC>1.2) in the RV Endocardium of 5 controls pigs (T1 to T5) and of 7 rTOF pigs (F1 to F7). The red and green colors indicate relative transcript abundance (red = overexpressed, green = downregulated). The columns represent the 12 samples while the rows correspond to the 153 probes. Samples were classified using hierarchical clustering, according to similarity in change in relative transcript abundance.

Out of the 43,603 probes represented on the Sus Scrofa microarray (Agilent), 153 probes were significantly different in rTOF pigs versus controls, after preprocessing and statistical analysis (fold change>1.2; p<0.05) (Fig 1). These 153 probes correspond to 69 annotated genes with 54 HUGO genes (other than LOC) (S1 Table) implicated in different processes, notably heart failure markers genes, calcium handling and contractile function-related genes, electrophysiology and arrhythmia-related genes, cytoskeleton/cellular organization-related genes, that may be of particular interest for our model.

**Fig 2:**
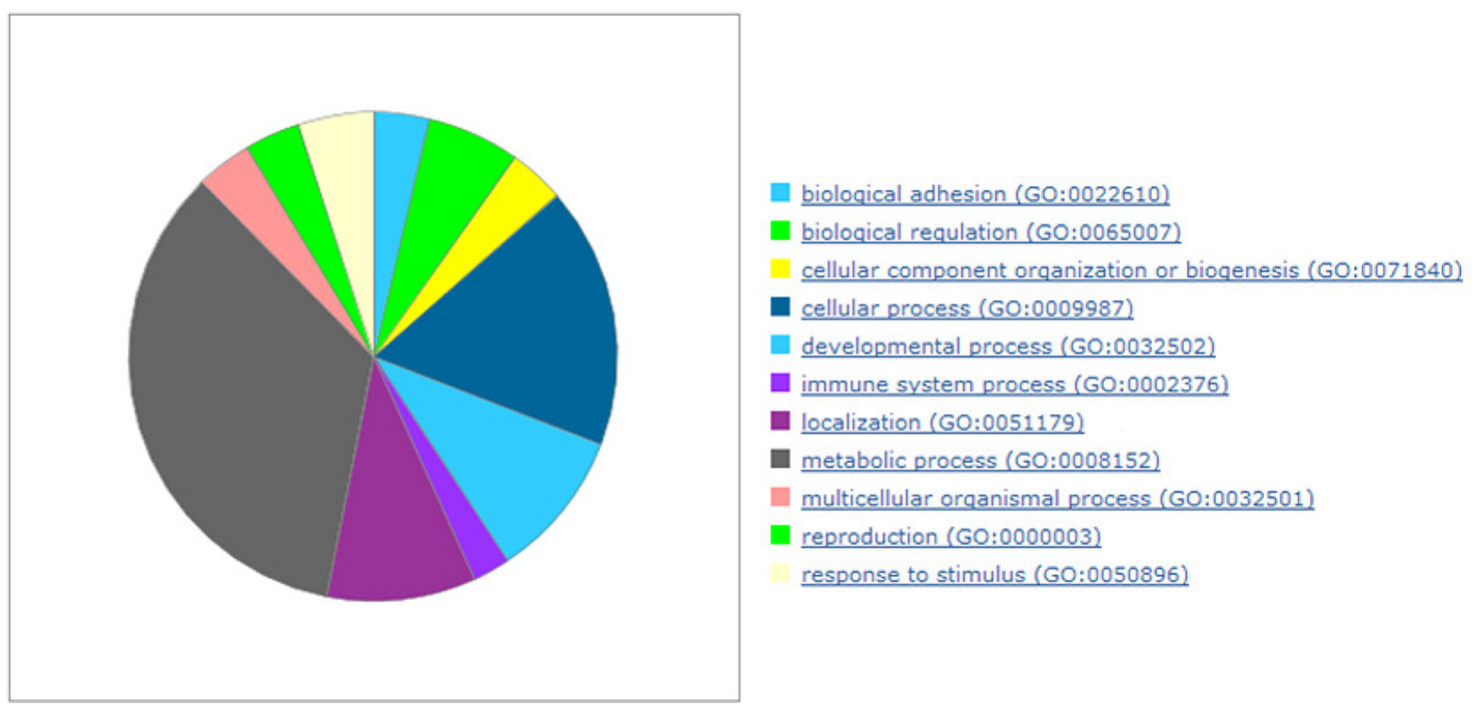
The significant gene ontology biological processes in the RV Endocardium.

The biological processes depicting genes that were deregulated in rTOF hearts are shown in Fig 2. “Metabolic process” (GO:0008152) and “Cellular process” (GO:0009987) are the most represented processes.

### Functional Pathway and Network Analysis

**Fig 3:**
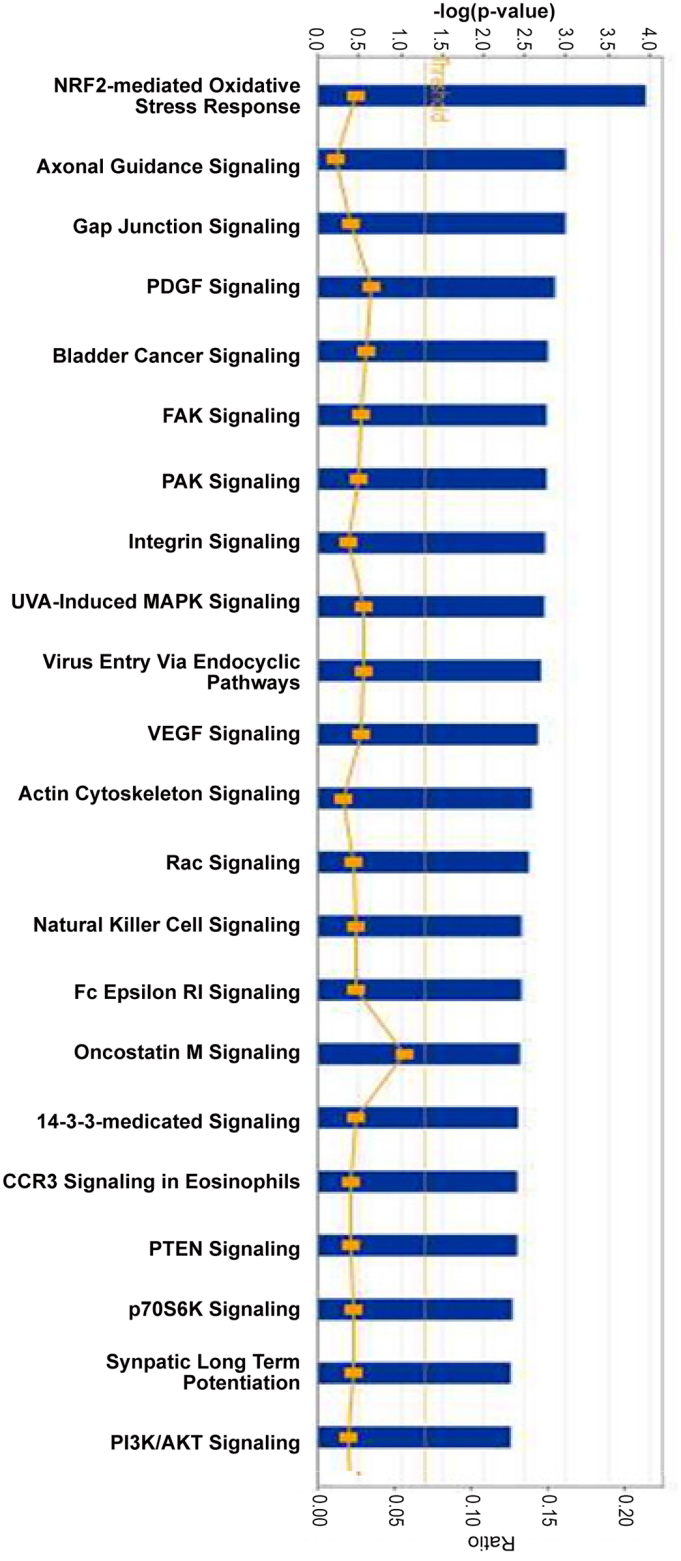
Functional categorization analysis of the most significant pathways represented in the microarray-generated list of the 54 most differentially expressed genes (Ingenuity software) in the RV Endocardium of rTOF pigs. The p-values (blue bars) were calculated using the right-tailed Fisher’s exact test. The threshold (yellow line) is set to a p-value = 0.05. The ratios (yellow dots and curve) represent the number of molecules of the experiment related to the number of total molecules involved in the pathways.

To investigate the interconnectivity of the differentially regulated genes with other gene products, pathways, and biological processes, molecular networks were formed with IPA software. These networks include genes from our transcriptomic data and their interactions with genes that are biologically relevant to the pathway, coding for hub molecules not altered in our experiments. Fig 3 shows the 22 most significant canonical pathways related to the 54 DE HUGO genes. Among these signaling cascades, the first is the NRF2-mediated Oxidative Stress Response that triggers apoptosis and necrosis. Then, the gap junction signaling related to electrical impulse propagation, the actin cytoskeleton signaling mediating cell motility and cell reshaping in response to extracellular stimuli, are the canonical pathways mostly involved. Other signaling pathways are linked to angiogenesis (PDGF and VEGF signaling), and to cellular adhesion (FAK signaling). The genes involved in these canonical pathways are listed in S2 Table.

**Fig 4:**
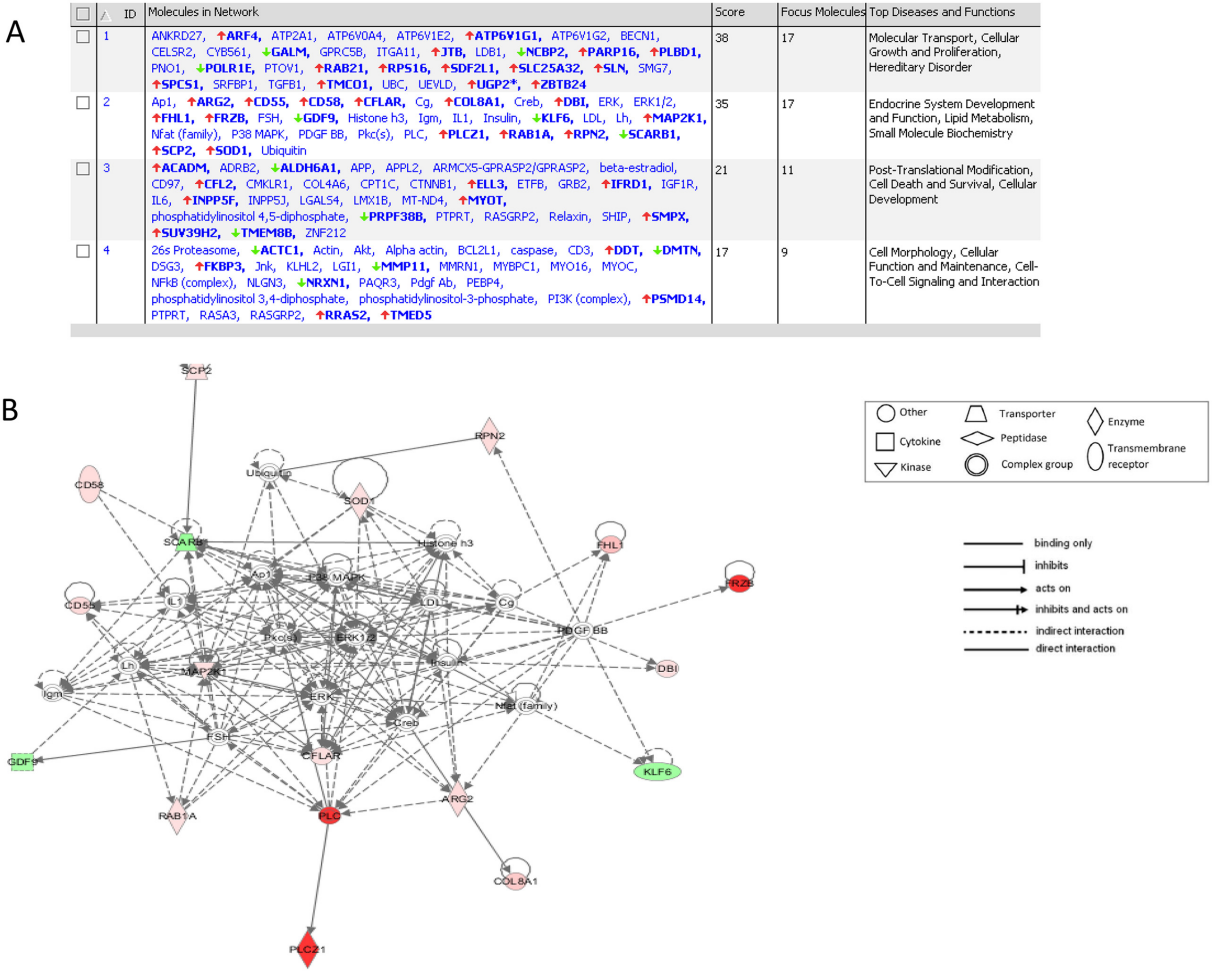
A. Ingenuity Pathway Analysis (IPA) generated 4 molecular networks assembled from DE genes the RV endocardium of rTOF pigs versus Controls (genes in bold) in the RV Endocardium. B. IPA Network #2. based upon differentially expressed genes that were upregulated and down regulated in the RV Endocardium.

The top 4 networks among the genes most differentially expressed are represented in Fig 4A. Each network was identified based on a numerical rank score according to the degree of relevance of the network to the molecules of our genes set and based on the hypergeometric distribution calculated as–log (Fisher’s exact test result). Genes involved in “Molecular transport, Cellular Growth and Proliferation” and “Endocrine system development and Function, Lipid Metabolism” were significantly overrepresented among differentially expressed genes. Fig 4B displays the network #2 with the highest number of genes of interest. In this network, ERK-PKC-MAPK are central hub molecules.

### Altered expression of genes implicated in different processes

**Fig 5:**
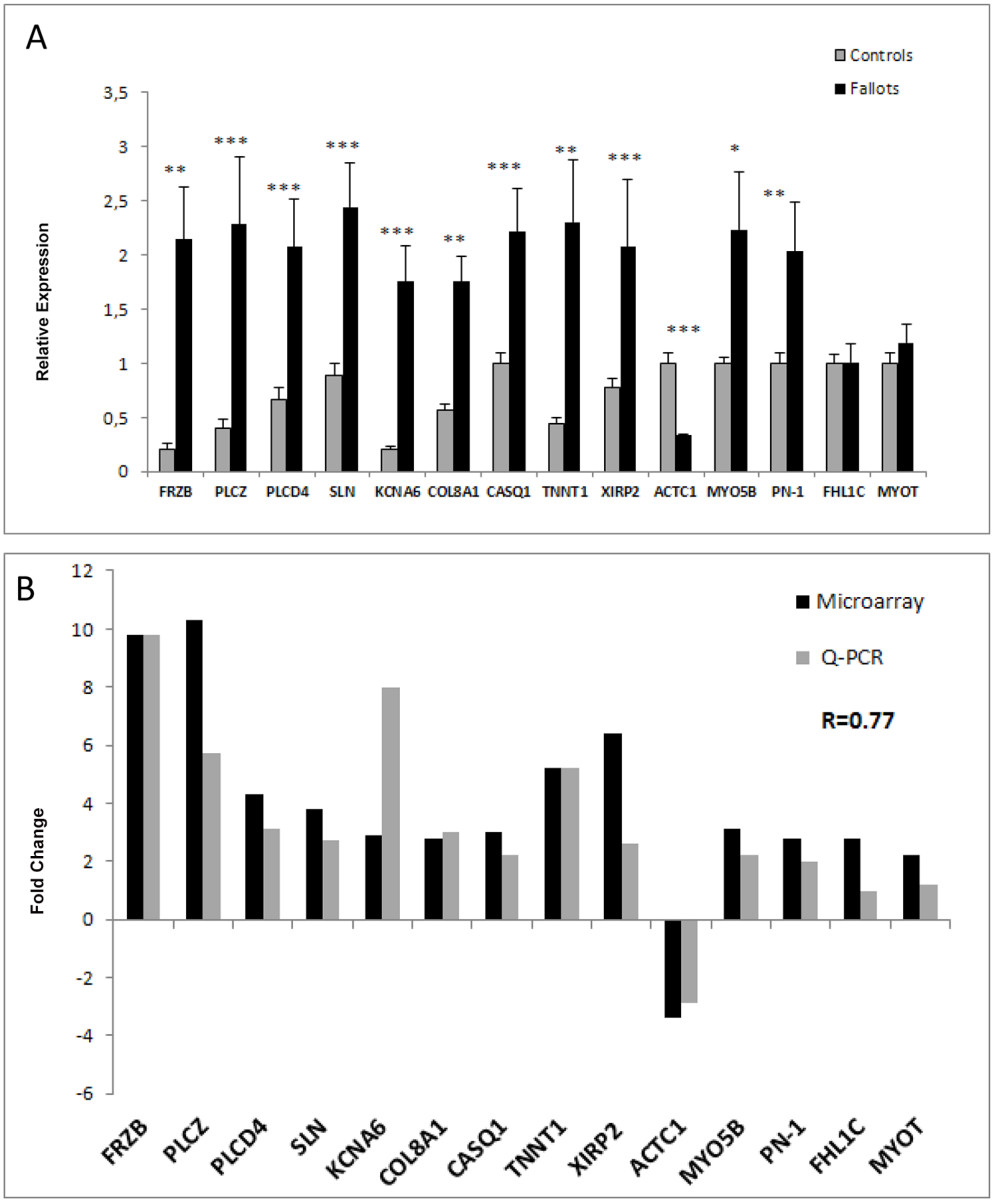
A. Relative expression (RT-qPCR) of genes in samples from the RV Endocardium of controls (grey bars) and rTOF (black bars) hearts. Transcript expression is normalized to the reference genes HPRT1 and GUSB. Two sided T-Test Statistical significance of n = 5 Controls and n = 7 rTOF hearts (*P <0.05, **P <0.01, ***P <0.001) B. Fold change comparison in samples from the RV Endocardium (n = 5 Controls and n = 7 rTOF hearts) based on RT-qPCR results and microarray expression data. Pearson correlation coefficient R = 0.77 (p = 0.001).

Specifically, relative to controls, rTOF pigs changed expression of heart failure markers genes—Secreted frizzled-related protein 1 (*FRZB*), calcium handling and contractile function-related genes—Sarcolipin (*SLN*), Alpha cardiac actin (*ACTC1*), Calsequestrin (*CASQ1*), Troponin (*TNNT1*), Myotilin (*MYOT*), Phospholipase C Z(*PLCZ*), Phospholipase C D4 (*PLCD4*), electrophysiology and arrhythmia-related genes—Myosin 5B (*MYO5B*), Four and a half LIM domains protein 1 (*FHL1C*), Collagen type VIII (*COL8A1*), Potassium voltage-gated channel subfamily A member 6 (*KCNA6*) and Cytoskeleton/Cellular organization-related genes—Xin actin-binding repeat-containing protein 2 (*XIRP2*), Nexin (*SERPINE2*) (Fig 5A).

### Validation of Gene Expression Pattern from Microarray Data Using qRT-PCR

Representative genes were selected for validation via qRT-PCR, using the same tissue samples used in the microarray. For these fourteen genes, the fold changes obtained via microarray analysis demonstrated substantial agreement with the fold change values determined via qRT-PCR (Pearson correlation coefficient R>0.7, p<0.05) thereby confirming the microarray data (Fig 5B). Two genes were no longer significantly deregulated using qPCR: *FHL1C* and *MYOT*. This could be due to different isoforms not tested by qPCR (9 targets deregulated in microarray for each of these two genes).

### Altered expression of other candidate genes

**Fig 6:**
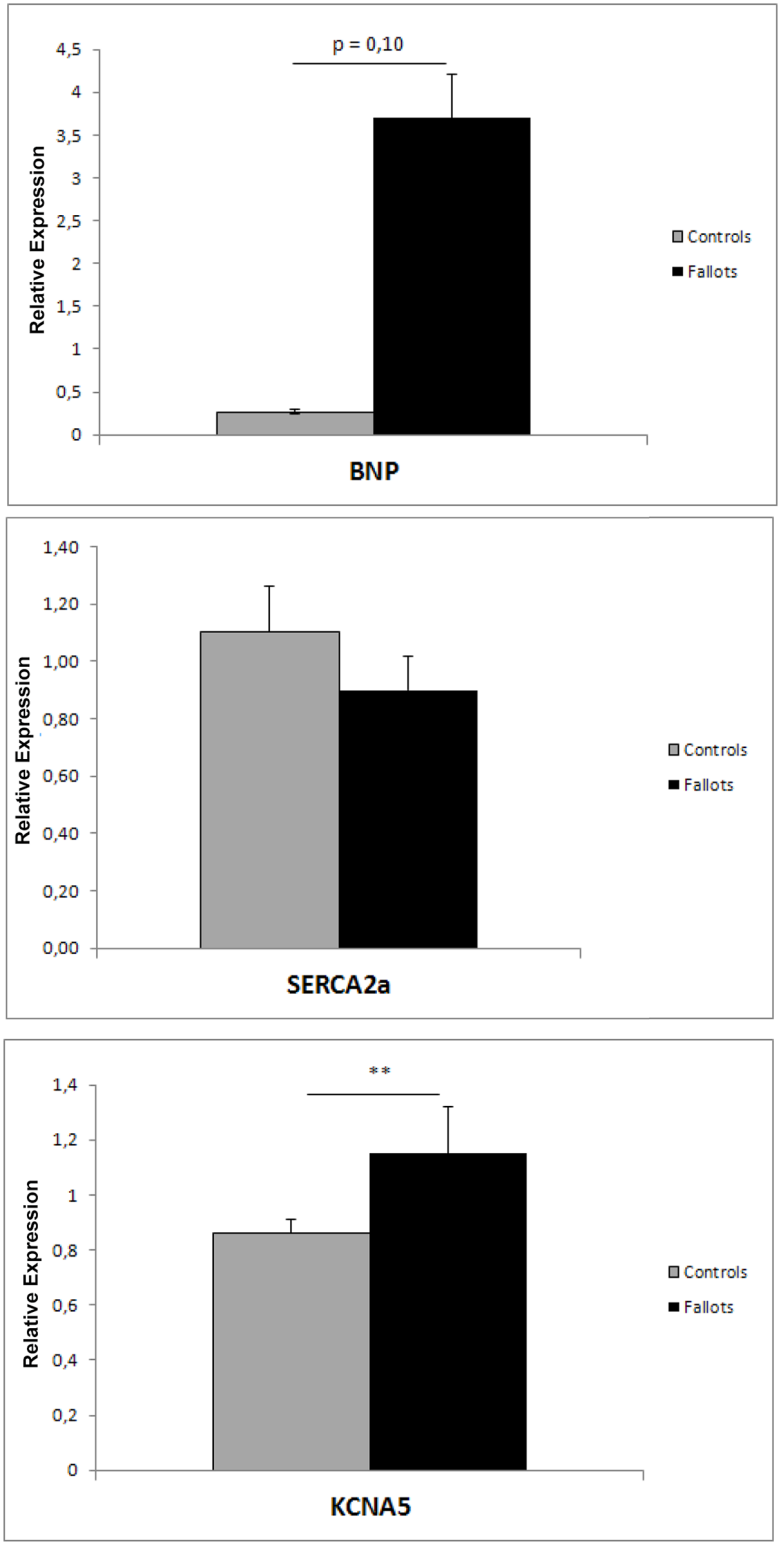
Relative expression (RT-qPCR) of *BNP*, Serca2a (*ATP2A2*) and *KCNA5* genes in samples from the RV Endocardium of controls (grey bars) and rTOF (black bars) hearts. Transcript expression is normalized to the reference genes HPRT1 and GUSB. Two sided T-Test Statistical significance of n = 5 Controls and n = 7 rTOF hearts (*P <0.05, **P <0.01, ***P <0.001).

We aimed to explore expression changes in candidate genes: *BNP* (Brain Natriuretic Peptide) as a heart failure marker, *SERCA2A2* (Sarcoplasmic/endoplasmic reticulum calcium ATPase 2, *ATP2A2*), known to be down regulated in failing hearts, and *KCNA5* coding for Kv1.5 which seems to interact with MYO5B and FHL1C (found deregulated in our microarray data) (Fig 6). We observed a statistically significant upregulation of *KCNA5* in the rTOF pigs compared to controls, and a trend to downregulation of *ATP2A2* in the rTOF pigs compared to controls. We observed a trend to upregulation of *BNP* (p = 0,10), however one of the 7 rTOF animals had a much stronger expression of *BNP* compared to others (S2 Fig). This rTOF pig also had one of the largest RV end-diastolic volume (data not shown).

### Expression Profiling in four different regions of the myocardium

Performing the same experiments and statistical analyses on the three other localizations (right ventricle epicardium, left ventricle endocardium and left ventricle epicardium), we found a marked heterogeneity of regional gene expression. Target genes were globally differentially expressed in the same way as in the RV endocardium, between the rTOF and the controls, but with no statistical significant p-value (p>0.05). Table 1 shows the fold change and p-values for 14 genes of interest chosen from the RV endocardium transcriptomic data, for the three other locations.

**Table 1:** Fold change comparison between samples from the RV endocardium (rTOF (n = 7)r versus controls (n = 5)), RV epicardium (rTOF (n = 4) versus controls (n = 4)), LV epicardium (rTOF (n = 4) versus controls (n = 4)), and LV endorcadium (rTOF (n = 7) versus controls (n = 5)) based on microarray expression data of 14 genes.

Gene Symbol	Description	RV Endo	RV Epi	LV Epi	LV Endo
*PLCZ*	Phospholipase C (Zeta)	10.3 (0.025)	5.3 (0.378)	1.5 (1.000)	2.0 (0.675)
*FRZB*	Secreted frizzled-related protein	9.8 (0.042) *	17.4 (0.378)	4.4 (1.000)	2.3 (0.528)
*XIRP2*	Xin actin-binding repeat-containing protein 2	6.4 (0.003) **	2.1 (0.542)	-1.3 (1.000)	0.9 (0.915)
*TNNT1*	Troponin	5.2 (0.023) *	3.4 (0.429)	2.7 (1.000)	2;0 (0.511)
*PLCD4*	Phospholipase C D4	4.3 (0.031) *	2.2 (0.378)	1.,3 (1.000)	1.5 (0.858)
*SLN*	Sarcolipin	3.8 (0.034) *	1.9 (0.588)	2.2 (1.000)	-1.2 (0.858)
*MYO5B*	Myosin	3.1 (0.004) **	2.0 (0.438)	2.9 (1.000)	2.9 (0.315)
*CASQ1*	Calsequestrin	3.0 (0,004) **	2.4 (0.378)	1.4 (1.000)	1.3 (0.793)
*KCNA6*	Potassium voltage-gated channel subfamily A member 6 (Kv1.6)	2.9 (0.028) *	8.5 (0.378)	2.0 (1.000)	4.0 (0.361)
*COL8A1*	Collagen type VIII	2.8 (0.040) *	1.09 (0.79)	-1.32 (1.000)	-1.27 (0.75)
*FHL1C*	Four and a half LIM domains protein 1	2.8 (0.037) *	1.9 (0.400)	1.2 (1.000)	1.3 (0.511)
*PN-1*	Nexin	2.8 (0.006) **	1.7 (0.452)	1.3 (1.000)	1.1 (0.922)
*MYOT*	Myotilin	2.2 (0.042) *	1.6 (0.468)	1.0 (1.000)	1.3 (0.616)
*ACTC1*	Actin	-3.4 (0.040) *	-1.6 (0.536)	-1.6 (1.000)	-1.4 (0.717)

Statistical significance (*P <0.05, **P <0.01, ***P <0.001).

**Fig 7:**
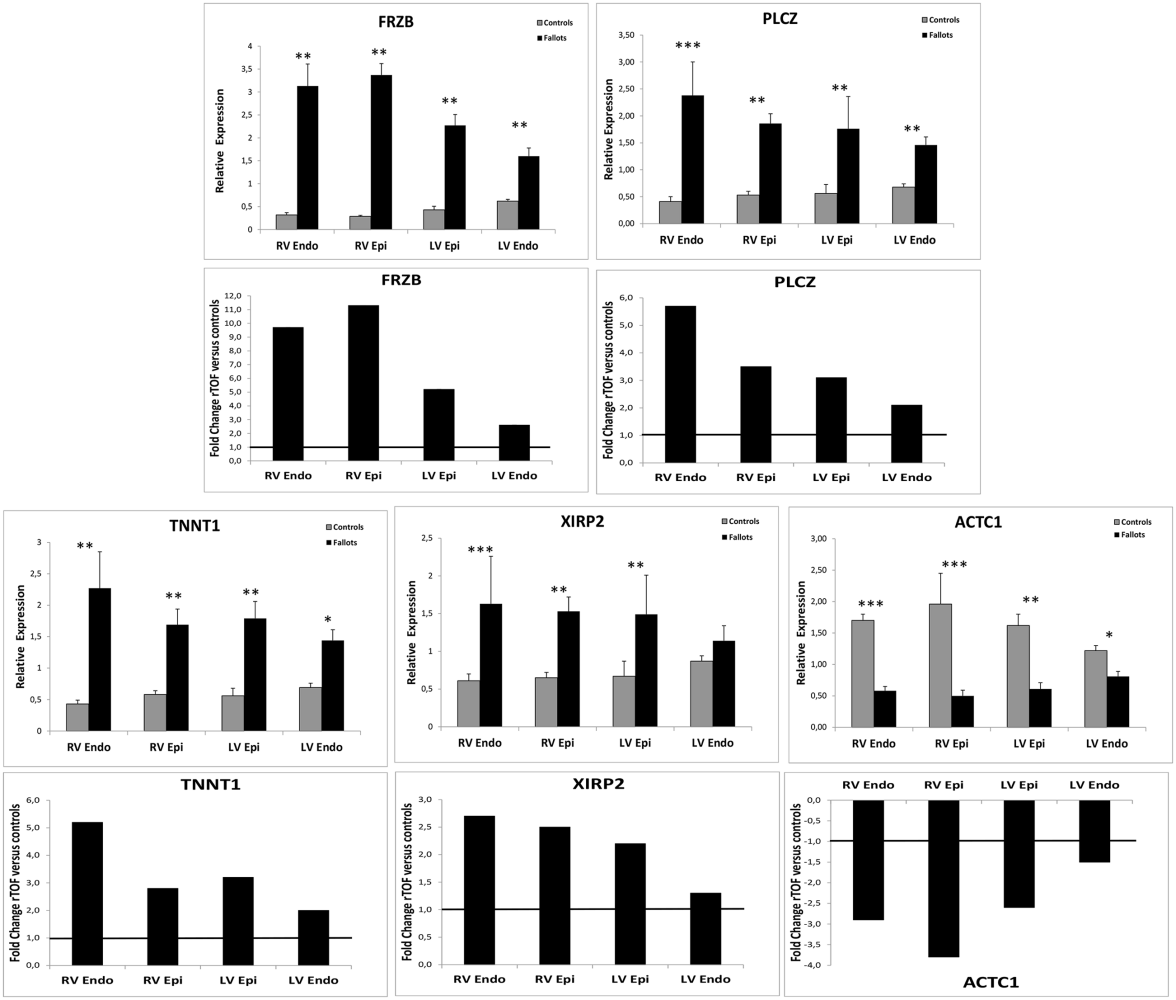
Relative expression (RT-qPCR) of genes in control (open bars) and Fallots (closed bars) pigs’ hearts and Fold change comparison between samples of the four localizations. Transcript expression is normalized to the reference genes HPRT1 and GUSB. Two sided T-Test Statistical significance of n = 5 Controls and n = 7 rTOF hearts (*P <0.05, **P <0.01, ***P <0.001).

We then used a candidate gene approach and studied, by quantitative RT-PCR, samples from the four localizations by testing 5 genes: *FRZB*, *PLCZ*, *TNNT1*, *XIRP2* and *ACTC1* (Fig 7). Surprisingly, we observed significant deregulation of these genes in the four localizations but with a lower FC. This could be due to the much higher sensitivity of qPCR compared to microarray.

## Discussion

To our knowledge, this study represents the first characterization of myocardial transcriptomic profiles in a long-term swine model of surgically repaired Tetralogy of Fallot. Not surprisingly, the most statistically differentially expressed genes were identified in the endocardium of the right ventricle, as expected in a model of RV primitive pathogenesis.

According to Panther Pathway software, the analysis of the 54 most DE genes in the RV endocardium revealed two main biological processes: cellular process and metabolic process. Ingenuity Pathway software pointed out the most significant canonical pathways including oxidative stress, energy metabolism, cell communication (gap junction signaling), cell adhesion and reshaping in response to external stimuli, angiogenesis. Molecular networks were also formed with IPA software involving the lipid metabolism that may reflect the changes in energy metabolism associated with ventricular hypertrophy. In this network, ERK-PKC-MAPK are central hub molecules. The role of the pathway MAPK/ERK/PKC in cardiac hyperthrophy has been well established [9,10].

Differentially expressed genes identified by microarrays were further validated using RTqPCR. We confirmed the level of transcript abundance of fourteen genes of interest using RT-qPCR, and the Fold Changes were concordant in most cases. Among the most de-regulated genes in the endocardium of right ventricle, we identified some genes of special interest.

### Heart failure markers

The BNP (Brain Natriuretic Peptide) is a cardiokine that also belongs to the fetal cardiac gene program and is used as a biomarker of cardiac hypertrophy and heart failure in humans because it is secreted in response to myocardial stretching. As expected, its expression tended to be upregulated among our rTOF pigs. The *FRZB* gene codes for a secreted frizzled-related protein B expressed in ventricular myocardium and involved in the Wnt/β-catenin signaling cascade affecting cell proliferation signals and endocardial cushion morphogenesis [11]. This gene was found overexpressed in human failing ventricular myocardium linked to overload-associated myocyte apoptosis [12]. Its overexpression observed in our rTOF pigs is in accordance with these previous findings and confirm FRZB as a marker of cardiac failure due to overload. Of note, myocardial mRNA levels (and serum levels) of a cardiokine called Frizzled-related protein 3, were found elevated in patients with end-stage HF (levels correlated with HF severity and BNP dosage)[13]. These observations may also make these secreted proteins new potential therapeutic targets.

### Calcium handling and contractile function genes

The SERCA2a protein (encoded by *ATP2A2)*, is a key regulator of intracellular Ca2+ trafficking, pumping it back to Sarcoplasmic Reticulum during myocardial relaxation. It is also a member of the fetal cardiac gene program which is reactivated with cardiac hypertrophy. This protein tended to be down-regulated in the RV of the rTOF pigs compared to controls (even if the difference was not significant), as it was observed in human RV endomyocardial biopsies at early stage of the Fallot disease (in immature cardiomyocytes with hypertrophy and hypoxia from children aged 15 to 29 months) [14]. This downregulation of *ATP2A2* is typical of heart failure, and was also recently described in a rat model of right heart failure [15]. Studies from Sharma or Ronkainen also pointed out a downregulation of *ATP2A2* in the cardiomyocytes of hypoxia-induced hypertrophic RV [16,17].

*SLN* coding for sarcolipin also belongs to the Ca2+ regulatory protein family. It is predominantly expressed in the cardiac atria in humans, however there are chamber specific and species specific differences in its expression [18]. SLN and PLN (phospholamban) are two small structurally similar proteins that inhibit SERCA2a and regulate cardiac contractility [19]. The increased expression of *SLN* in our model represents a new finding compared to the study by Wu et al. that did not find any deregulation of this gene at the RNA and protein level in human cardiomyocytes, and compared to other studies studying SLN under several pathological conditions [14,19]. Interestingly, Sarcolipin was found up-regulated 50 fold in the hypertrophied ventricles of Nkx2.5 null mice [20]. Mouse models overexpressing SLN had reduced SERCA2a activity and *in vivo* measurements of cardiac function showed a significant decrease of +dP/dt and–dP/dt with ventricular hypertrophy. The inhibitory effect of SLN on these models was reversed by a βadrenergic agonist, isoproterenol, which restored cardiac contractility [21].

The absence of concomittant deregulation of *PLN* (data not shown) could argue for a possible independent role of SLN in inhibiting SERCA2a, as previously hypothesized [19].

*ACTC1*, coding for a cytoskeletal protein or alpha cardiac actin, was the most significantly downregulated gene in our set and was already described as associated with the pathogenesis of dilated cardiomyopathy and heart failure in several expression studies [22]. Autosomal dominant mutations in *ACTC1* were found in familial Atrial septal Defect and reduced expression of *ACTC1* was observed in different congenital heart diseases in humans including Tetralogy of Fallot [23]. Our data could suggest that this gene was more linked to the adaptative ventricular remodeling than to the genetically determined congenital cardiac malformation.

*CASQ1* was found significantly up regulated in the rTOFs, however this isoform is predominantly expressed in the skeletal muscle in humans and pigs (*CASQ2* being the main cardiac isoform).

Phospholipases C D4 and Z were found upregulated in the rTOF pigs confirming the link of phospholipase C with PKC / Frizzled / Wnt signaling pathway [24].

### Electrophysiology and arrhythmia-related genes

Myosin 5B coding for the unconventional myosin motor VB (among 3 members A, B, C of the class V of the myosin superfamily) is overexpressed in our model. It is a molecular motor that was recently found to regulate the cell surface trafficking of ion channels, such as Kv1.5 (*KCNA5* gene) and control channel recycling in rat cardiomyocytes [25]. The *KCNA5* gene was also significantly upregulated in the right ventricles of our rTOF pigs. This gene, mainly expressed in the atrium, mediates IKur and contributes to atrial repolarization. It has also been shown to be expressed in the human left ventricle [26] and would be functional in canine ventricular myocytes [27]. It was observed in mice ventricular myocytes, contributing to repolarization [28]. However, its contribution to repolarization in human or pig ventricles remains to be demonstrated.

*FHL1C* was a good candidate gene as this protein, predominantly expressed in skeletal and cardiac muscle, is implicated in several hereditary myopathies and was also shown to interact *in vitro* with the voltage-gated Potassium channel Kv1.5 [29]. However its overexpression found in microarrays was not confirmed with qPCR. Maybe this could be due to the existence of different isoforms of the gene. Further experiments are needed in order to explore this hypothesis.

### Cytoskeleton/Cellular organization-related genes

*KCNA6* (encoding Kv1.6 channel) is significantly expressed in human cardiac fibroblasts [30] while its expression has never been described in human or pig ventricular myocytes. Moreover, in the mouse, undifferentiated cardiac c-kit (+) cells, Kv1.6 was shown to participate in regulating cell proliferation [31]. Thus, the increase in *KCNA6* expression we observed in our animal model may reflect fibroblast proliferation.

*XIRP2* (overexpressed in rTOF pigs) belongs to the evolutionarily conserved, muscle specific, actin-binding Xin gene family. This protein (mXinβ) has a role in the intercalated disc maturation and post-natal heart growth in mice [32]. This gene was up regulated in several *in vitro* and *in vivo* models of hypertrophy and heart failure at early stage [33]. XIRP2 appeared to be an essential mediator of angiotensin II-induced pathological cardiac remodeling, a direct transcriptional target of Angiotensin II signaling pathway in cardiac muscle, through MEF2A (myocyte enhancer factor), involved in cell survival pathway in cardiac stress signaling [34,35].

Interestingly, *COL8A1* was found significantly activated in our rTOF group as it was already observed in the cardiac RV of a mouse model of pulmonary artery clipping [36]. This collagen, upregulated in rTOF pigs, seems to be associated with arterial stiffness [37] but could also be a marker of RV remodeling.

### Regional variations in gene expression

Transcriptional responses in the left ventricle in our set of experiments followed the same tendency than for the right ventricle, but were much less strong. Comparison of the expression levels of different genes (*TNNT1*, *FRZB*, *ACTC1*, *PLCZ* and *XIRP2)*, in all four locations, led to hypothesize the existence of a molecular “gradient” of expression progressing from the RV to the LV of rTOF pigs. Mechanical stress could be transmitted from RV to LV through molecular and cellular alterations mediated by transcriptomic changes of certain genes. The nature and extent of gene expression vary with time. Indeed, distinct transcriptional changes were observed at different time-points after pulmonary artery clipping surgery in a mouse model [36]. Therefore it will be of interest to perform transcriptomic studies at different stages post-surgery and study the progression of the molecular remodeling in our animal model.

### Limitations

mRNA deregulations may not always reflect abnormal protein levels, since translational processes and post-translational modifications may occur. We performed some protein studies by western blot in order to confirm the mRNA changes at the protein level: for example TNNT1 protein tend to be upregulated in the rTOF pigs, as observed in mRNA studies (S3 Fig). Studies on phosphorylation of different proteins will also be of interest in order to decipher post-translational changes already observed in cardiac failure.

Moreover, the incomplete annotation of the *sus scrofa* microarray constitutes a limitation of this study as important genes, not present or not annotated in the chip, could be missed and signaling pathways of interest could be incompletely deciphered. This led us to have a complementary target gene approach by performing RT-qPCR directly on other genes of interest.

## Conclusion

Cardiac studies performed on pigs proved to be of interest as large animal models have a closer physiology to humans than small mammal models. Moreover, use of cDNA microarrays is a good and cost-effective tool to identify new candidate genes by systematic exploration of gene expression on a genome-wide scale. By providing wide information it should permit potential comparisons among different models and contribute to a greater understanding of the molecular mechanisms linked to cardiac disease.

## Supporting Information

S1 Fig Final day cardiac function evaluation in Control and Fallot pigs. Anesthetised pigs underwent cardiac magnetic resonance examination at 23 ± 1 weeks. A: Cardiac equatorial short axis view of a Fallot pig showing RV hypertrophy, dilation and septal bulging. B: RV end-diastolic volume (RVEDV) was significantly increased in Fallot pigs compared to Control indicating RV dilatation. C: RV ejection fraction (RVEF) was significantly lower in Fallot pigs than Controls highlighting RV dysfunction. Control N = 4, Fallot N = 6, * P < 0.05; *** P< 0.001.
(TIF)

S2 Fig BNP gene expression in Rv endocardium. Relative expression (RT-qPCR) of BNP gene in samples from the RV endocardium of controls and rTOF hearts. Transcript expression is normalized to the reference genes HPRT1 and GUSB.
(TIF)

S3 Fig TNNT1 Western blot. 50μg of total protein were loaded for TNNT1 Western blot analysis. Quantification of band intensity was calculated after normalization for total protein loaded (p = 0.08).
(TIF)

S1 Table List of 54 HUGO genes differentially expressed in rTOF pigs (microarrays data). (TIF)

S2 Table List of genes involved in each of the 22 most significant canonical pathways (IPA). (TIF)
