# Supplementary figures and images for: Identification of Region-Specific Myocardial Gene Expression Patterns in a Chronic Swine Model of Repaired Tetralogy of Fallot
Source: PLoS One. 2015 Aug 7;10(8):e0134146. doi: 10.1371/journal.pone.0134146 (PMC4529093; doi:10.1371/journal.pone.0134146)

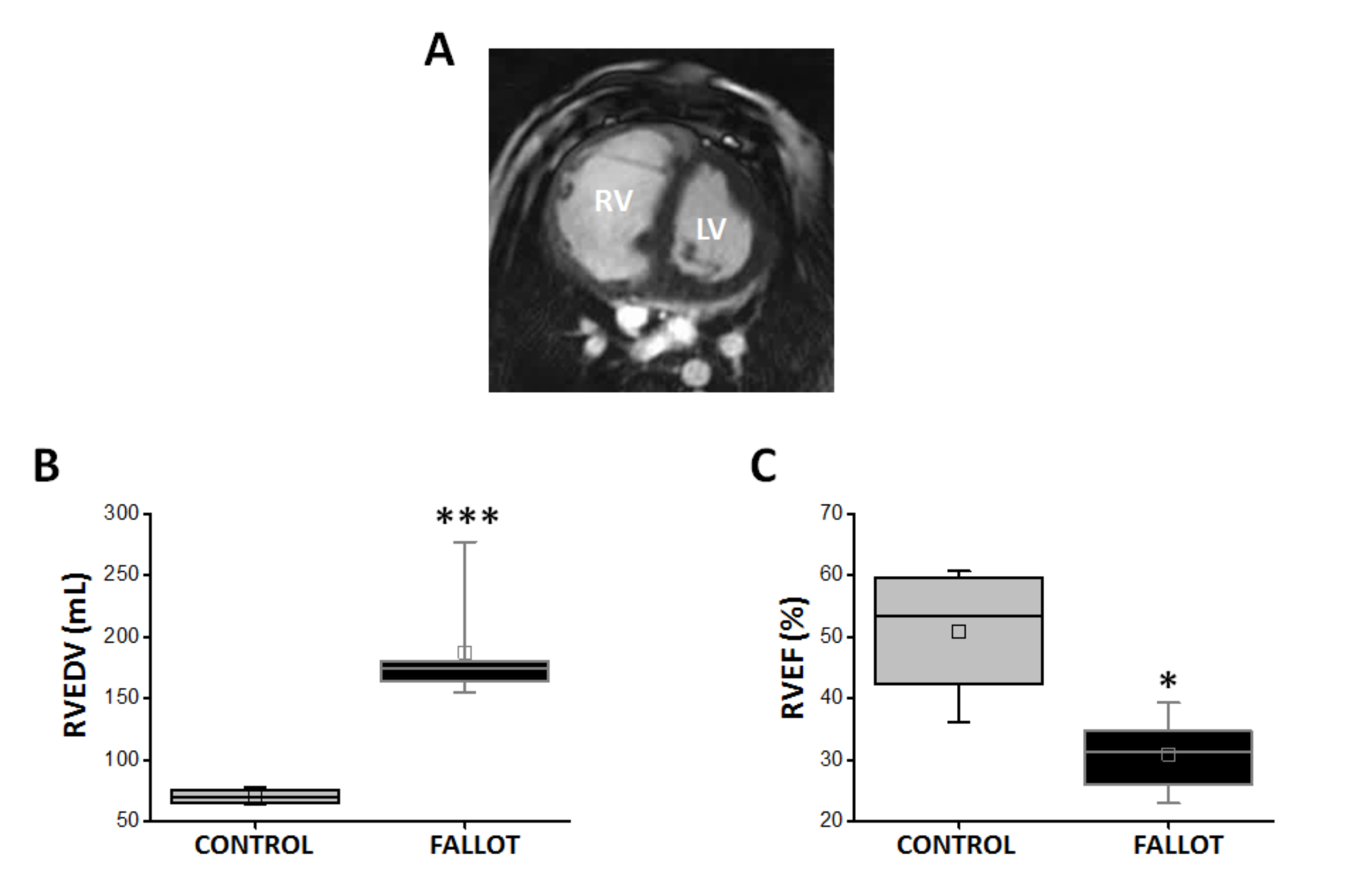

Supplement: S1 Fig — Anesthetised pigs underwent cardiac magnetic resonance examination at 23 ± 1 weeks. A: Cardiac equatorial short axis view of a Fallot pig showing RV hypertrophy, dilation and septal bulging. B: RV end-diastolic volume (RVEDV) was significantly increased in Fallot pigs compared to Control indicating RV dilatation. C: RV ejection fraction (RVEF) was significantly lower in Fallot pigs than Controls highlighting RV dysfunction. Control N = 4, Fallot N = 6, * P < 0.05; *** P< 0.001. (TIF) [file pone.0134146.s001.tif]

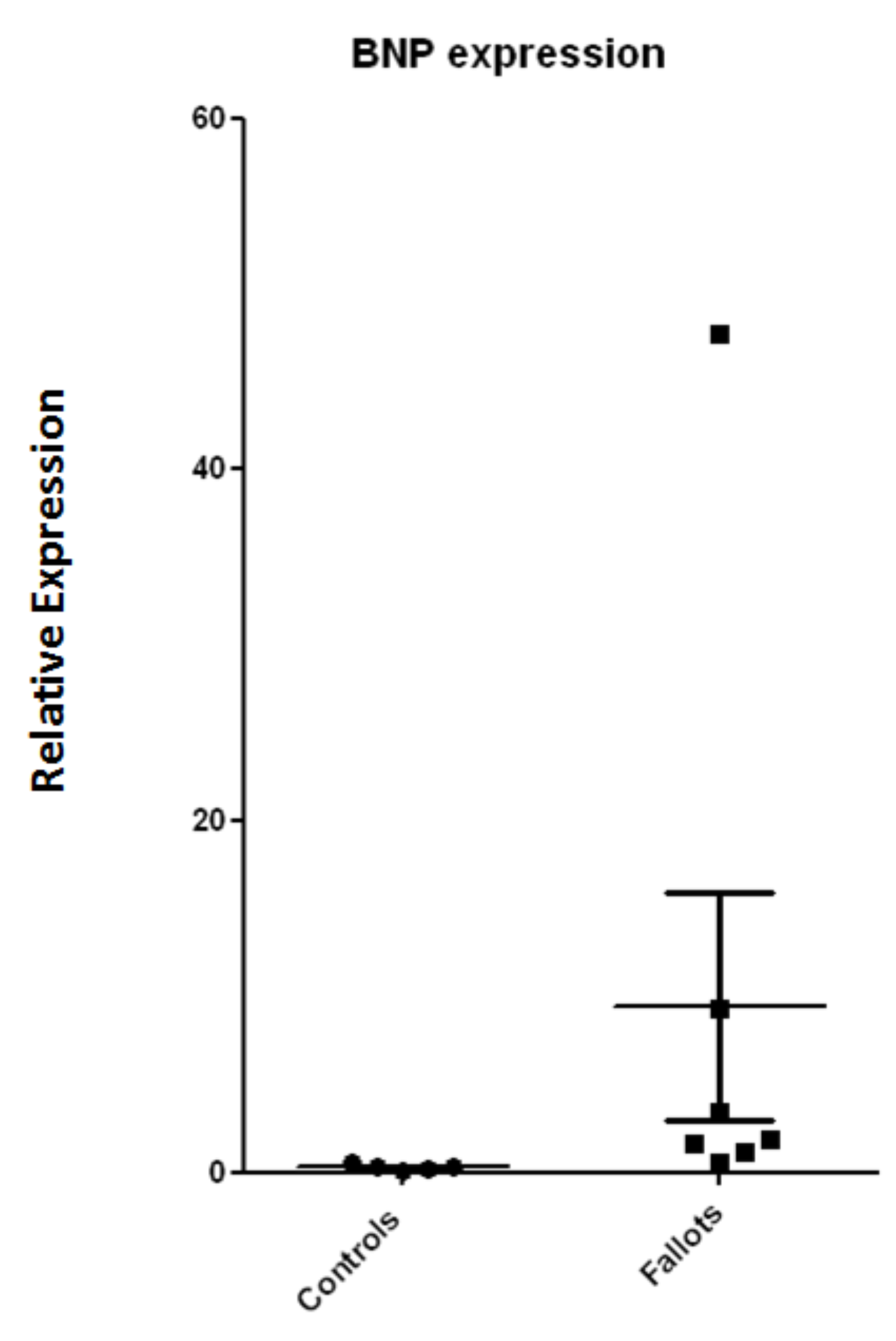

Supplement: S2 Fig — Relative expression (RT-qPCR) of BNP gene in samples from the RV endocardium of controls and rTOF hearts. Transcript expression is normalized to the reference genes HPRT1 and GUSB. (TIF) [file pone.0134146.s002.tif]

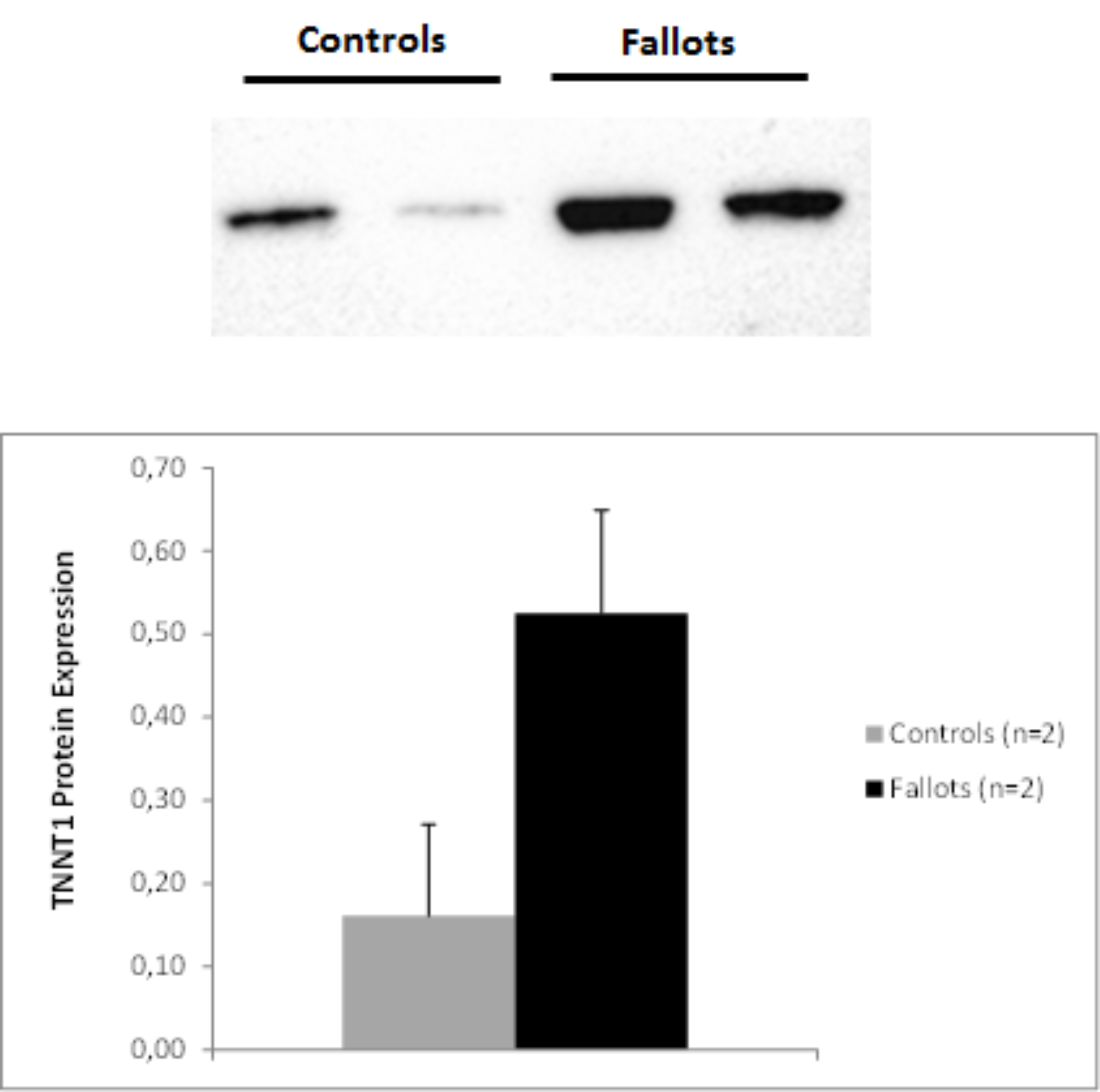

Supplement: S3 Fig — 50μg of total protein were loaded for TNNT1 Western blot analysis. Quantification of band intensity was calculated after normalization for total protein loaded (p = 0.08). (TIF) [file pone.0134146.s003.tif]

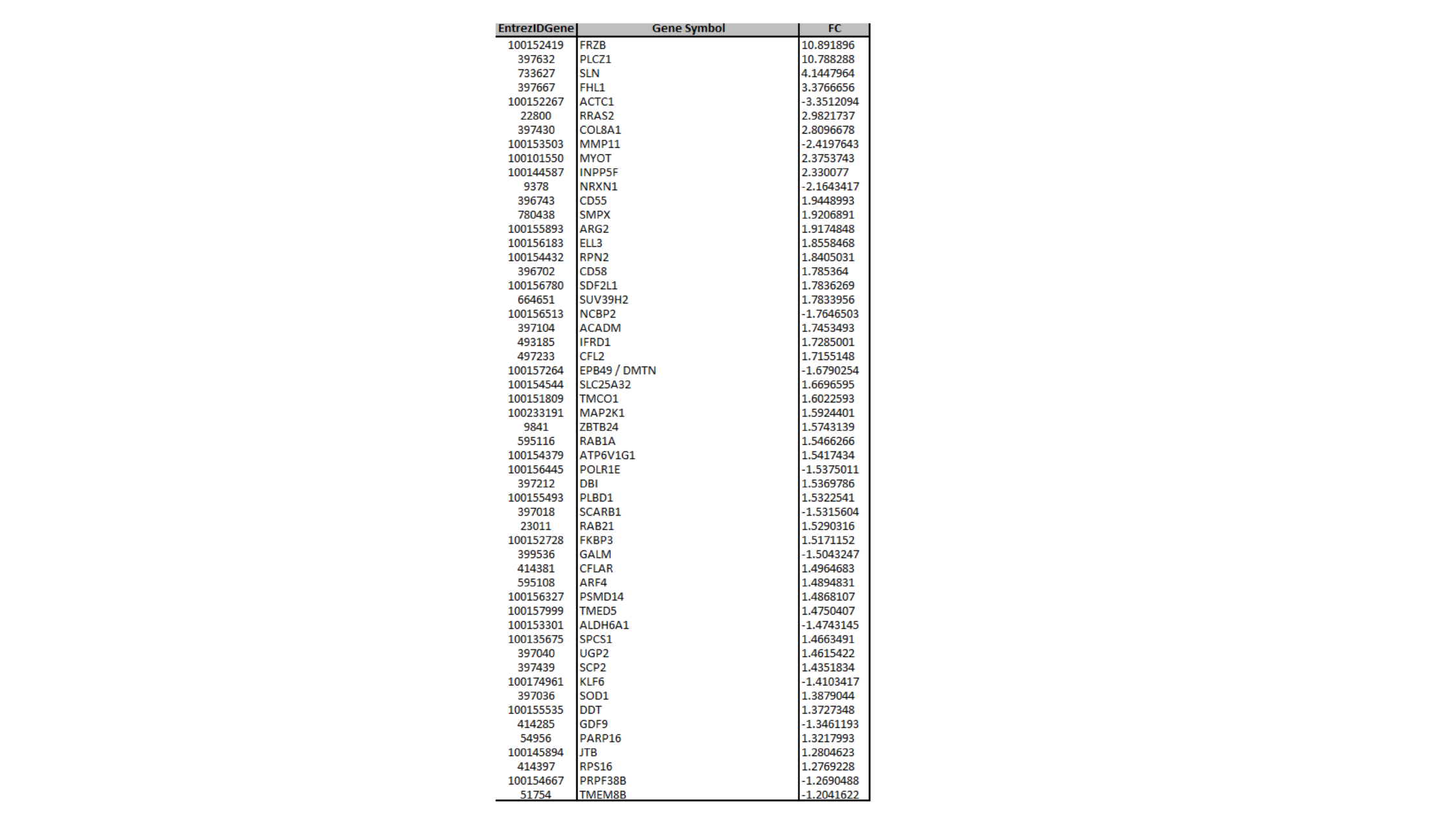

Supplement: S1 Table — (TIF) [file pone.0134146.s004.tif]

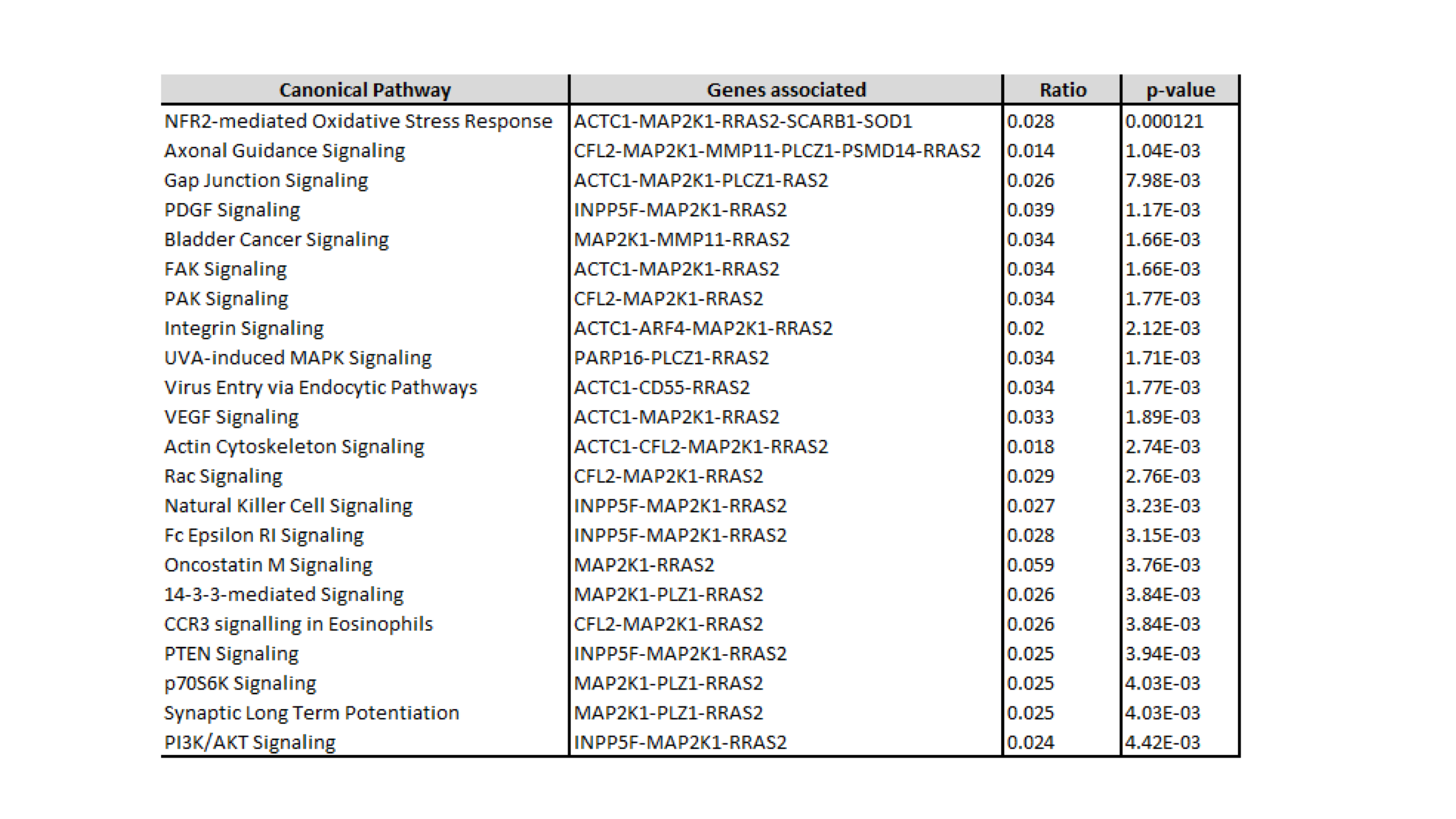

Supplement: S2 Table — (TIF) [file pone.0134146.s005.tif]
